# Impact of teaching on use of mechanical chest compression devices: a simulation-based trial

**DOI:** 10.1186/s12245-024-00611-7

**Published:** 2024-02-26

**Authors:** Richard Steffen, Simon Burri, Fredy-Michel Roten, Markus Huber, Jürgen Knapp

**Affiliations:** 1 Department of Anaesthesiology and Pain Medicine, Inselspital, Bern University Hospital, University of Bern, Bern, 3010 Switzerland; 2 Swiss Air Rescue, Rega, Zurich, Switzerland; 3 Valais Cantonal Rescue Organization, Sierre, Switzerland

**Keywords:** Cardiopulmonary resuscitation, Mechanical chest compression devices, Medical education, Simulation

## Abstract

**Background:**

The use of mechanical chest compression devices on patients in cardiac arrest has not shown benefits in previous trials. This is surprising, given that these devices can deliver consistently high-quality chest compressions without interruption. It is possible that this discrepancy is due to the no-flow time (NFT) during the application of the device. In this study, we aimed to demonstrate a reduction in no-flow time during cardiopulmonary resuscitation (CPR) with mechanical chest compression devices following 10 min of structured training in novices.

**Methods:**

270 medical students were recruited for the study. The participants were divided as a convenience sample into two groups. Both groups were instructed in how to use the device according to the manufacturer’s specifications. The control group trained in teams of three, according to their own needs, to familiarise themselves with the device. The intervention group received 10 min of structured team training, also in teams of three. The participants then had to go through a CPR scenario in an ad-hoc team of three, in order to evaluate the training effect.

**Results:**

The median NFT was 26.0 s (IQR: 20.0–30.0) in the intervention group and 37.0 s (IQR: 29.0–42.0) in the control group (*p* < 0.001). In a follow-up examination of the intervention group four months after the training, the NFT was 34.5 s (IQR: 24.0–45.8). This represented a significant deterioration (*p* = 0.015) and was at the same level as the control group immediately after training (*p* = 0.650). The position of the compression stamp did not differ significantly between the groups. Groups that lifted the manikin to position the backboard achieved an NFT of 35.0 s (IQR: 27.5–42.0), compared to 41.0 s (IQR: 36.5–50.5) for the groups that turned the manikin to the side (*p* = 0.074).

**Conclusions:**

This simulation-based study demonstrated that structured training can significantly reduce the no-flow time when using mechanical resuscitation devices, even in ad-hoc teams. However, this benefit seems to be short-lived: after four months no effect could be detected.

**Supplementary Information:**

The online version contains supplementary material available at 10.1186/s12245-024-00611-7.

## Introduction

For the outcomes of patients in cardiac arrest, high-quality cardiopulmonary resuscitation (CPR) is of paramount importance. This includes high-quality chest compressions, as well as interruptions of the chest compressions that are kept as short as possible [1]. During manual CPR, compressions must be interrupted not only for defibrillation and rhythm analysis, but also to replace fatigued rescuers. Mechanical chest compression devices (CCD) can shorten these pauses and thus increase the fraction of chest compressions [2]. Nonetheless, three large randomised controlled trials including more than 11,000 patients did not show any benefits from automated mechanical CCDs over manual chest compressions in out-of-hospital cardiac arrest (OHCA) [3–6].

One possible reason for this finding is that a negative outcome after OHCA is associated with the duration of the single longest pause in chest compressions, and in most OHCA with the use of mechanical devices, the longest pause was the one for device application [2, 7]. We therefore hypothesised that enhanced training quality, as well as repeated training, is needed to reduce no-flow time (NFT) during application of a CCD.

## Materials and methods

This study was conducted between October 2021 and March 2022 at the Bern University Hospital. In their third academic year, all medical students at the University of Bern are required to participate in a basic life support (BLS) course, in accordance the European Resuscitation Council (ERC) guidelines. As a result, prior to taking this course most participants have only marginal knowledge of CPR.

Participation in this study was voluntary and free of charge. We obtained written consent to use the data from all participants, who were assured that their performance in the study scenario would have no influence on their course result. All 270 students agreed to participate in this study. The cantonal ethics committee of Bern confirmed that there was no authorisation requirement (Req-2021-01026, 09/07/2021).

### Study setting and equipment

We used a standardised adult simulation manikin for CPR training (Resusci Anne®, Laerdal Medical, Norway) for training and assessments. The mechanical CCD used in this study was a proprietary Lund University Cardiac Assist System (LUCAS-3®, Physio Control, Redmond, Washington, USA). These devices deliver chest compressions via a compression stamp and a battery-powered piston mechanism. A board must be placed under the patient’s back to provide counter support.

The application of the CCD by the participants was recorded on video. We carried out these recordings with two wide-angle cameras (ZOOM© Q2n-4 K). One camera was positioned at the foot of the mannequin and the other one beside the head. This setup ensured a clear view of the CCD at all times, enabling the NFT to be assessed correctly. Additionally, the manikin’s feedback software was used to evaluate the duration of interruption of chest compressions. The participants were blinded to the endpoints of the study.

### Training and assessment

The participants were instructed in BLS following the guidelines of the European Resuscitation Council (ERC) and trained in teams of three. In conformity with the guidelines, the importance of high-quality chest compressions with minimal interruptions was emphasised several times during BLS training. All participants were additionally trained in the use of the mechanical CCD. To avoid possible bias due to conversations among participants, they were divided into two groups as a convenience sample: participants during the first half of the semester (October to early November 2021) were included in the control group, while participants during the second half of the semester (November to December 2021) were assigned to the intervention group. Both groups were introduced to the use of CCD via the manufacturer’s training videos. Subsequently, in the control group the participants were given 10 min to familiarise themselves with the device and train individually in their teams according to their own needs. The teams could choose between lifting the upper body of the manikin (“lifting method”) or turning it to one side (“rolling method”), in order to position the back-board under the body (both techniques were demonstrated in the manufacturer’s video). The instructor only answered technical questions and assisted the participants with handling issues such as how to click the device onto the plate or the positioning of the stabilisation straps. The instructor did not comment on medical or theoretical content. In contrast, the intervention group underwent 10 min of structured team training, in which the participants had to apply the resuscitation device three times in a short sequence as part of short CPR scenario. The position of the individual subjects (for airway management, chest compressions and the application of the resuscitation device) had to be rotated during each scenario of the training session. Thus, the training given to the intervention group went more in the direction of “drill and practice”, in the sense of a repetitive and disciplined exercise. In this group, we focused not only on training participants to handle the CCD, but on the importance of teamwork when putting on the device. As part of the structured and more standardised training mode in the intervention group, only the lifting method was taught during this training session. As with the control group, the instructors only answered technical questions here and had no influence on the content and process of the training. They only ensured compliance with the training schedule (changes of position of the participants) and time management.

Before the end of the BLS course, we asked participants to use the resuscitation device during an ongoing resuscitation in a simulated clinical scenario (evaluation scenario). The task was set up using a short introductory video. The participants completed the scenario in teams of three, with each participant selected from a different group than the one from the BLS course. Thus, the participants had never before used the resuscitation device in this particular group configuration. The resuscitation device was provided to the group after the first resuscitation cycle, and the scenario was terminated as soon as the device was able to deliver adequate chest compressions.

The participants from the intervention group were invited to a follow-up study four months later. The same task was set as for the assessment at the end of the BLS course, and we used the same video to introduce the CPR scenario. Since we used the no-flow times of the control group as a baseline, a follow-up examination of this group was deliberately omitted.

### Outcome measures

The primary endpoint was the cumulative no-flow time (NFT). All interruptions between the arrival of the CCD and the definitive takeover of the chest compressions by the device were added up. We used video analysis conducted by two independent investigators to measure these times, which were then confirmed by recordings from the manikin’s feedback software. Secondary endpoints included the correct positioning of the compression stamp of the CCD (deviation of +/- 40 mm is assumed as the limit for correct positioning), while NFT was compared between the different techniques for positioning of the backboard.

### Statistical analysis

This study was designed as a pragmatic study, and participants were recruited as part of their CPR training course. As a result, the size of the group was predetermined. While power analysis was deliberately omitted due to this setting, effect sizes were statistically examined during the analysis. Summary measures for all outcomes are shown by means of the median and interquartile range (IQR) for continuous variables, and by counts and percentages for binary variables. Group comparisons were performed by means of the Mann-Whitney test for continuous variables and by means of the chi-square test or the exact Fisher test. All computations were performed with R version 4.0.2. We chose a significance level of 0.05 for all statistical tests.

## Results

We evaluated 37 teams in the control group, 49 teams in the intervention group, and 14 teams in the follow-up (Supplementary Fig. 1). As the number of participants in certain teaching groups was not divisible by three, some teams had to work with participants who had already gone through the scenario before. These teams were completely excluded from the analysis (9/109; 8%).

In the control group, the median NFT was 37.0 s (IQR: 29.0–42.0 s) compared to 26.0 s (IQR: 20.0–30.0 s) in the intervention group (*p* < 0.001, Table 1). By the time of the follow-up examination of the intervention group four months after the training course, the NFT had increased significantly compared to the resuscitation scenario immediately following the course (34.5 s, IQR: 24.0–45.8, *p* = 0.015), and was equal to that of the control group immediately after the training (37.0 s, IQR: 29.0–42.0 s, *p* = 0.650, Table 1).

**Table 1:** Primary outcome (median and interquartile range)

	Control Group	Intervention Group	Follow-up
	*n* = 37	*n* = 49	*n* = 14
**Cumulative no-flow time** (sec)	37.0 [29.0;42.0]	26.0 [20.0;30.0] *	34.5 [24.0;45.8] **

* *p* < 0.001 (compared to control group); ** *p* = 0.650 (compared to control group)

The device was correctly positioned in 89% of the control group and in 88% of the intervention group (*p* = 0.852). Thirty teams in the control group chose the lifting method, and 7 teams turned the manikin to the side to position the back-board of the CCD (rolling method). The lifting method showed a median NFT of 35.0 s (IQR: 27.5–42.0), compared to 41.0 s (IQR: 36.5–50.5) for the rolling method (*p* = 0.074).

## Discussion

In recent years, enormous efforts have been made in ALS and BLS training to minimise pauses in chest compressions, especially before and after defibrillation attempts [8, 9]. However, to our knowledge, no study has been conducted on how to reduce NFT during the application of a mechanical CCD. Our results show that structured team training in the installation of a CCD significantly decreased NFT in a simulated scenario of CPR. Replacing the standard instructional briefing and 10 min of training with the device according to the individual needs of the participants with 10 min of structured team training shortened the NFT for the application of the device by a median of 11 s. While there exists an abundant literature promoting intensive training for the use of AED, for example, our study is the first to highlight the importance of structured team training, not only for the standard ALS sequence, but also for the integration of a mechanical CCD into this algorithm [10, 11]. During the evaluation scenario, the teams were composed of participants from different groups from the BLS training course, meaning that the participants had never used a CCD together in this configuration before. This indicates that structured team training for CCD application is sustainable even in situations with changing teams composed on an ad-hoc basis, as is often the case in the everyday work of emergency medicine. We deliberately chose a team size of three members, because this seemed to represent a realistic situation: In the Swiss EMS, two paramedics usually work together as the crew of an ambulance. In most cases of OHCA, an EMS doctor or an advanced paramedic is also called to the scene. Even if only a two-person team is primarily on the scene, in most cases a qualified lay assistant or relative can carry out sufficient chest compressions under the guidance of the rescue team.

However, four months after the introductory training, the effect vanished and the interventional group needed the same amount of time to apply the CCD as the control group. This demonstrates the need for repeated, rigorous training with this device. Although the importance of repeated training in BLS, ALS and the use of defibrillators is widely known, our study provides evidence that training in the application of a CCD should also be repeated at regular intervals [12–16].

Three large randomised controlled trials comparing mechanical chest compression with manual chest compression have shown no benefit for mechanical CCD. This seems surprising, as especially during prolonged resuscitations, manual chest compressions are given at incorrect rates or depth, and are interrupted for frequent and lengthy pauses [17–19]. Mechanical CCD would address all these problems and substantially improve the quality of chest compressions. However, the interruption of chest compressions during the application of a CCD might counteract these benefits. These pauses for device application were recorded only in a small subgroup of patients (*n* = 67) in one of the above-mentioned trials, the LINC trial: the median was 36.0 s (IQR: 19.5–45.5 s). This closely aligns with the results of our control group (37.0, IQR: 29.0–42.0), in which the participants were instructed and trained in the use of the CCD, but did not receive the structured team training. In 42 of these 67 records in the LINC trial (63%), this pause for device application was the longest NFT during CPR [2]. As survival rate after cardiac arrest seems to be associated with the duration of the single longest pause in chest compression, this might not only partly explain the negative results in the previous trials, but also underline the clinical significance of a structured team training on the application of mechanical CCD [3–5]. However, even the improved NFT of 26 s in the intervention group is a long way from what we are aiming for. This study potentially identifies an important starting point for reducing NFT by at least 11 s: structured and repeated team training. The shockingly long time of 26 s even after optimised training or 37 s after conventional device instruction in our simulation-based study corresponds to reality, as shown, for example, by the results of the LINC trial, where NFTs of 36 s were measured. This reflects a typical problem in the context of optimising the care of patients in cardiac arrest. If measures are not thoroughly drilled in and lead to a prolongation of the NFT, such as tracheal intubation, patients are unlikely to benefit from them [20, 21]. The same applies to the use of point-of-care ultrasound during CPR [22, 23]. Moreover, these long NFTs also emphasise that, at the moment, CCD should be reserved for well-trained high-performance teams in a specific context. What our results also show, however, is that if even three helpers with optimised training currently require 26 s, the use of the CCD with only two helpers will lead to an even longer NFT and therefore cannot be supported. Nevertheless, there are situations in which the use of CCD (whether with two or three rescuers) is also indicated by the guidelines: prolonged cardiopulmonary resuscitation (e.g. hypothermia, severe hyperkalaemia, anaphylaxis and pulmonary embolism) and resuscitation at high altitude (as cardiopulmonary resuscitation is more exhausting for the rescuer than at sea level). That said, the use of CCD will gain particular importance in the future due to the increasing establishment of extracorporeal CPR (eCPR) in specialized centres – and therefore the need to transport patients under continuous chest compression [24–26].

Although only the “lifting method” was used in the structured training, all the teams were free to choose the method for placing the CCD. In the intervention group, only the “lifting method” was used, whereas, in the control group, 7 out of 30 teams preferred the “rolling method”. The “lifting method” seems to have a median time advantage of 6 s, which, however, was not significant. As this study was not intended to detect differences between these two methods, and this time difference would be of relevance in clinical practice, it seems worthwhile to investigate this issue in another trial. In this follow-up study, the weight of the manikin should equal to that of a real patient, and the influence of the patients’ clothes in positioning the back-board should be taken into account.

## Limitations

Our study has clear limitations. Naturally, the use of a simulated scenario can only partially reflect the conditions and relevant aspects of resuscitation (stress, environment, etc.). However, the advantage is that we were able to ensure standardised and comparable conditions for both groups. This study was designed as a pragmatic study. With a given group size, we were unable to carry out a power analysis. However, the effect size could be proven statistically. Furthermore, the correct position of the compression pad of the CCD was chosen arbitrarily, with a deviation of 40 mm. However, even in the international guidelines, the pressure point is not precisely specified [27]. Another possible limitation is that, in the intervention group, only the “lifting method” was trained and the choice of method in the control group was left open. However, NFT in the teams of the control group using the “lifting method” was 35.0 s [IQR: 27.5–42.0] compared to 26.0 s [IQR: 20.0–30.0] in the intervention group (*p* < 0.001). This seems to indicate a training effect rather than an effect due to the different methods of attachment. Finally, the participants’ effective experience in resuscitation was not systematically recorded. Nonetheless, it can be assumed that they were all novices, as medical students with existing experience (e.g. paramedics and nurses) were not required to participate in the BLS course.

## Conclusion

We were able to show that the change from the usual device training for mechanical CCD to a structured team training can significantly reduce the NFT. This effect was demonstrated in teams that had not trained together before. At the same time, we found that this benefit is short-lived: after four months the training effect seems to have vanished. This may indicate the importance of repetitive structured team training for all providers using these devices. Future studies must show whether patient outcomes can be improved through better training in application of CCD.

### Electronic supplementary material

Below is the link to the electronic supplementary material.

Supplementary Material 1

## Data Availability

The datasets generated and/or analysed during this study can be obtained from the corresponding author on reasonable request.
