# Supplementary figures and images for: Impact of teaching on use of mechanical chest compression devices: a simulation-based trial
Source: Int J Emerg Med. 2024 Feb 26;17:26. doi: 10.1186/s12245-024-00611-7 (PMC10895751; doi:10.1186/s12245-024-00611-7)

**Supplemental figure 1**


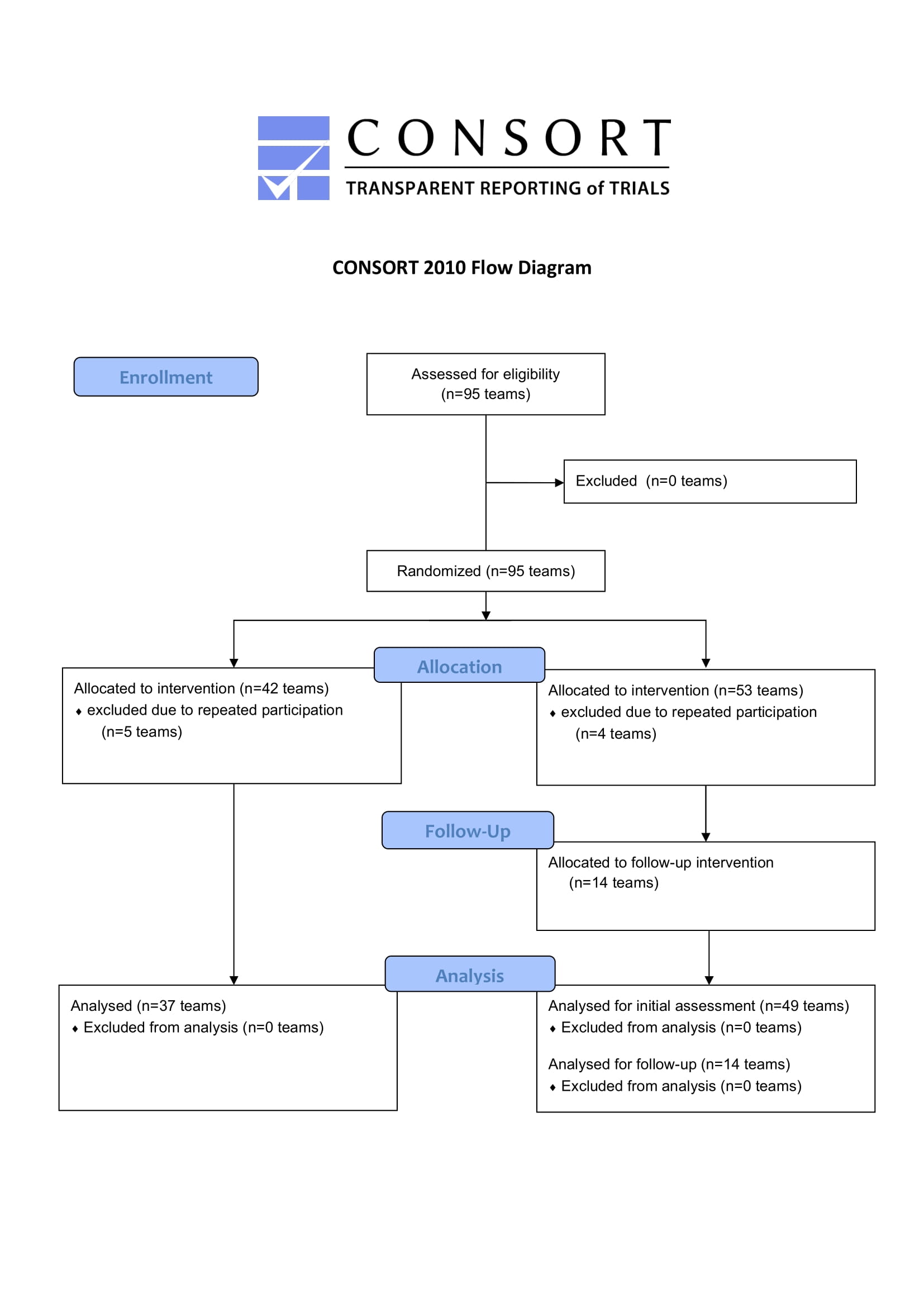

Supplement: Supplementary file 1 — Supplementary Material 1 [file 12245_2024_611_MOESM1_ESM.docx]
